# A bibliometric analysis of resistance to PD-1/PD-L1 inhibitors

**DOI:** 10.3389/fimmu.2026.1788252

**Published:** 2026-05-28

**Authors:** Lehan Miao, Zhendong Jiang

**Affiliations:** 1 The Fifth Clinical College, Zhuhai Campus of Zunyi Medical University, Zhuhai, Guangdong, China; 2 Department of Basic Education, Zhuhai Campus of Zunyi Medical University, Zhuhai, Guangdong, China

**Keywords:** bibliometric analysis, cancer, drug resistance, immunotherapy, PD-1/PD-L1

## Abstract

**Background:**

Programmed cell death protein 1 (PD-1) or Programmed death-ligand 1 (PD-L1) inhibitors have already reformed cancer treatment by inhibiting tumor immune escape. However, as drug resistance emerges, their clinical application is limited. Numerous studies have explored mechanisms and methods to address resistance, yet an explicit bibliometric analysis of this field remains lacking.

**Methods:**

We systematically retrieved documents from the Web of Science Core Collection database and Scopus database. The timeline spans from January 1, 2006, to November 29, 2025. A comprehensive analysis was conducted using bibliometric tools. In addition, a secondary screening of the included studies was conducted to classify and analyze them based on their experimental models.

**Results:**

The bibliometric analysis of 2,122 studies shows a significant increase in research on resistance to PD-1/PD-L1 inhibitors from 2006 to 2025. China leads in publication volume, while the United States ranks second but has more international collaboration. Institutions such as Memorial Sloan Kettering Cancer Center are at the forefront of this research. The *Journal of Clinical Oncology* and Stephen L. Topalian were the most influential journal and author in this field, respectively. Keywords and reference analysis identify prospective hotspots, including “tumor microenvironment”, “combination strategy”, and “biomarker”. Research on resistance to PD-1/PD-L1 inhibitors is primarily based on observational cohort studies.

**Conclusion:**

This study examines the academic background and hotspots in PD-1/PD-L1 resistance. In the future, the tumor microenvironment will be explored to uncover novel mechanisms of resistance and to identify predictive biomarkers for patient stratification, while combination therapy strategies will be applied to overcome therapeutic resistance. More randomized controlled trials (RCTs) and research on patient-derived organoid systems are needed to accelerate clinical translation. By illuminating future trends, this study will serve as a crucial reference for scholars seeking to advance the field.

## 1 Introduction

As the second leading cause of mortality across the world, cancer has seriously threatened current human and global public health (1). In 2023, Global estimates indicate an annual incidence of 18.5 million new cancer cases (Non-melanomatous skin cancers are excluded), alongside 10.4 million deaths attributable to cancer (2). The Global Cancer Observatory (GLOBOCAN) 2022 also shows that global cancer incidence has increased by more than 50% over the past three decades (3). It underscores the urgent need for precise and effective treatment strategies. At present, the primary modalities for cancer treatment remain surgery, chemotherapy, and radiotherapy, while gene therapy and immunotherapy are being increasingly integrated (4). Immunotherapy can enhance the intensity of the body’s immune response to tumor cells, thereby inhibiting or eliminating them. It has become one of the most widely studied cancer treatment strategies in recent years (5). Immune treatment research primarily includes immune checkpoint inhibitors, adoptive cell therapy, tumor vaccines, and related cytokine therapies (6). Among these, immune checkpoint inhibitors, particularly targeting the PD-1/PD-L1, have remarkable clinical efficacy in cancer treatment (7).

PD-1 is a primary co-inhibitory receptor expressed on activated T cells. When combined primarily with its ligand PD-L1, it will recruit the phosphatase Src homology region 2-containing protein tyrosine phosphatase 2 (SHP-2) to the vicinity of the T cell receptor (TCR) and CD28 signaling complexes, leading to the dephosphorylation of key signaling molecules (8). Consequently, T cell activation and cytotoxic function are suppressed, ultimately resulting in the death of the activated T cells (9). The PD-1/PD-L1 signaling pathway plays a significant role in limiting T cell responses and protecting tissues from autoimmunity. However, cancer cells can also express PD-L1, which can bind to activated T cells to evade immune surveillance. Immune checkpoint inhibitors can block tumor progression by inhibiting this process (10, 11). In 2014, the US Food and Drug Administration (FDA) approved the first PD-1 antibody drug (nivolumab) (12). Since then, the clinical adoption of monoclonal antibodies targeting PD-1/PD-L1 has become rapid and widespread, particularly in the treatment of non-small cell lung cancer, bladder cancer, melanoma, renal cell carcinoma, and Hodgkin lymphoma (13–17). However, Clinical trials have shown that some patients had no response to anti-PD-1/PD-L1 at the start due to primary resistance (18). A more retrospective study showed that 61% of non-small cell lung cancer cases developed acquired resistance after treatment, and half (52%) of them progressed within 1 year (19). Tumor treatment resistance remains a key obstacle in clinical oncology (20). It poses a significant challenge for cancer immunotherapy by undermining the therapeutic efficacy of PD-1/PD-L1 inhibitors (21). Numerous studies have summarized resistance mechanisms and corresponding strategies. However, the existing literature may fail to provide researchers with a systematic understanding of the research landscape, the evolution of hotspots, and future directions. Therefore, quantitative synthesis and visual mapping are essential for distilling complex literature into intuitive insights.

Bibliometric analysis, the quantitative appraisal of scholarly literature, has emerged as an indispensable instrument for delineating the trajectory of scientific progress and identifying research gaps (22, 23). This study presents a comprehensive bibliometric evaluation of the research landscape of PD-1/PD-L1 inhibitor resistance from 2006 to 2025. We analyze global publication trends, identify key contributions from countries, institutions, journals, and authors, and explore the conceptual evolution of the field through citation bursts and keyword dynamics. In addition, we provide a systematic classification of existing research models and discuss prospective therapeutic strategies for overcoming resistance to PD-1/PD-L1 inhibitors. By providing multi-dimensional insights, this work aims to identify current limitations in the clinical translation of research and outline future directions for overcoming the therapeutic challenge.

## 2 Methods

### 2.1 Database and data filtering

**Figure 1:**
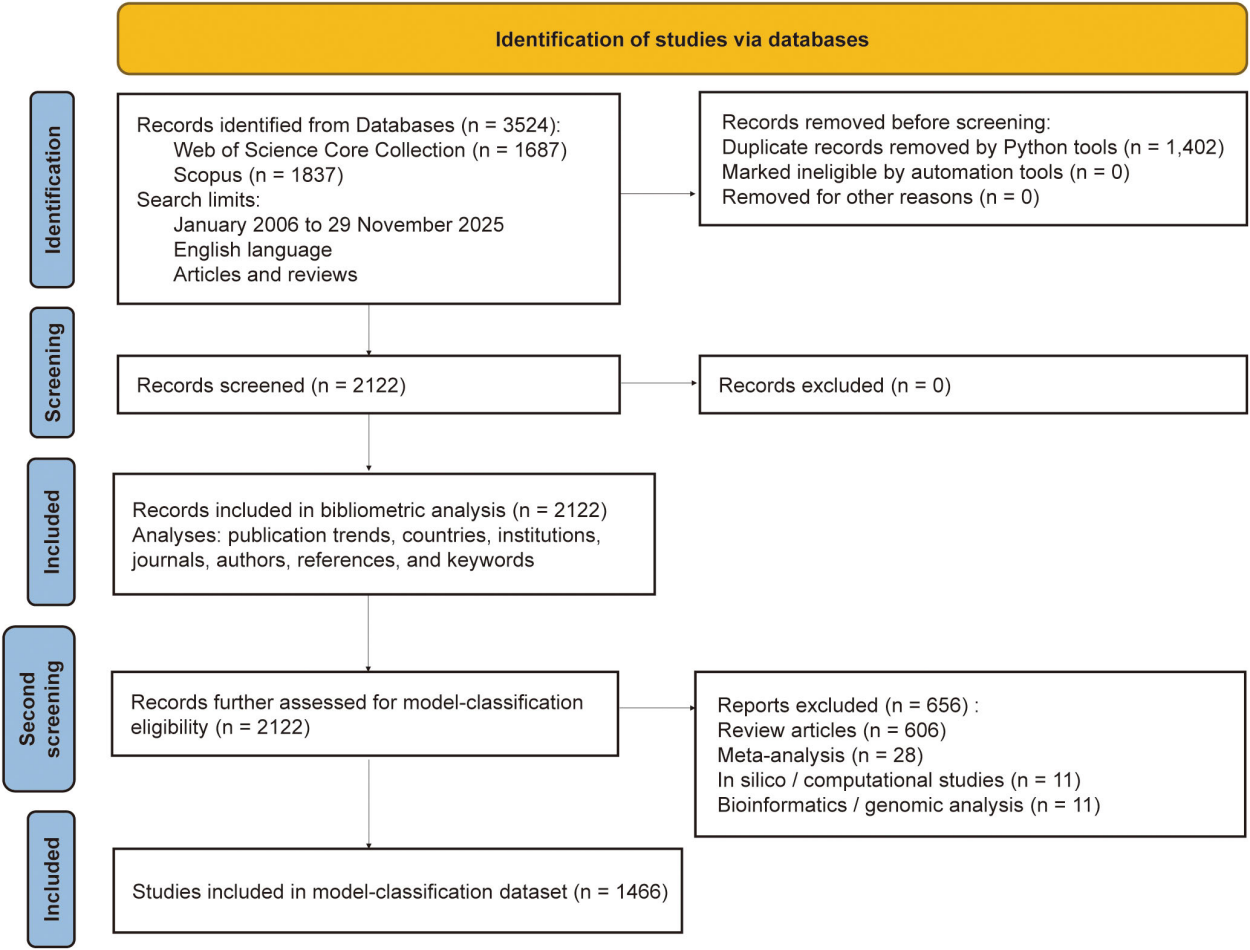
Flowchart of the search process.

We retrieved literature from the Web of Science Core Collection and Scopus databases. The search strategies for the two databases are listed in **Supplementary Table 1**. We initially screened the literature in the two databases by setting the conditions as follows: (1) Time span is set from January 1, 2006, to November 29, 2025. (2) The language is limited to English. (3) Only Articles and reviews were included (**Figure 1**). After the initial screening, we obtained 1,687 documents from WoSCC and 1,837 documents from Scopus. Using Python tools, we merged the documents and removed duplicates. During the deduplication process, records from WoSCC were prioritized for retention to ensure data integrity, given that they generally include more comprehensive bibliographic metadata than those from Scopus. Finally, 2,122 pieces of literature were retained and used for bibliometric analysis.

After the bibliometric dataset was constructed, a secondary eligibility assessment was conducted to derive the model-classification dataset. Because the model-classification analysis was designed to characterize experimental models used in original research, this stage included only studies that reported primary experimental data. Reviews, meta-analyses, purely in silico or computational studies, and purely bioinformatics or genomic analyses were excluded because they did not directly report the use of original experimental models. During this secondary screening, 606 reviews, 28 meta-analyses, 11 in silico/computational studies, and 11 bioinformatics/genomic analyses were excluded. Ultimately, 1,466 original studies were retained for model-based classification. Two researchers independently performed data merging and screening, and discrepancies were resolved through discussion until consensus was reached.

Because this study aimed primarily to map publication patterns, intellectual structures, and model-related research themes, PRISMA was used to ensure transparency in literature identification, screening, eligibility assessment, and dataset construction. The completed PRISMA 2020 checklist is provided as **Supplementary Table 2**. Items related to risk-of-bias assessment, effect measures, quantitative synthesis, reporting bias assessment, and certainty of evidence were considered not applicable, as no intervention-effect synthesis or meta-analysis was conducted.

### 2.2 Data analysis

To process and analyze the bibliometric data, we utilized Python 3.14, Microsoft Excel 2021, Tableau Desktop (version 2025.2), Scimago Graphica (version 1.0.53), VOSviewer (version 1.6.20), CiteSpace (version 6.1.6R), and the R package Bibliometrix (version 4.4.2). We use Python tools to merge and filter data. Microsoft Excel was used to create a trend chart reflecting global production. Tableau Desktop and Scimago Graphica were used to visualize the geographical distribution of publications and national cooperation. CiteSpace, developed by Chaomei Chen, was used to visualize countries’ citation networks and generate keyword timeline maps. We also use it to detect citation bursts for references and keywords, facilitating the identification of evolving trends and key research themes within the field (24). VOSviewer, developed by van Eck and Waltman, was used to create detailed visualizations of collaboration and co-cited networks between institutions, journals, and authors. We also used it to visualize keyword co-occurrence (25). The R package Bibliometrix provides statistical tools for analyzing publication metrics (26). Together, these tools provided a comprehensive framework for mapping the intellectual structure of PD-1/PD-L1 inhibitor resistance research and identifying influential contributors, hotspot trends, and potential areas for future investigation.

In the model-classification analysis, studies were grouped according to the primary experimental model used to generate their main findings. Human clinical samples included studies based primarily on patients, clinical specimens, clinical trials, real-world cohorts, medical records, registries, or case-based observations. This category was further subdivided into randomized controlled trials, prospective studies, retrospective studies, and case series according to the reported study design. Mouse models comprised murine *in vivo* systems, including xenograft, syngeneic, orthotopic, genetically engineered, and patient-derived xenograft models. *In vitro* studies referred to work conducted primarily in cell lines, primary cells, immune-cell assays, co-culture systems, protein-binding assays, or other non-animal experimental systems. Other animal models included non-mouse *in vivo* systems, such as rats, zebrafish, dogs, chick embryos, or non-human primates. Organoid studies were defined as studies in which organoids, tumoroids, spheroids, or patient-derived organoids served as the principal experimental platform.

Two reviewers independently classified the eligible original studies according to a predefined model-classification codebook. The unit of analysis was the individual study rather than each model occurrence; therefore, each eligible study was counted only once. For studies that included multiple experimental models, the primary model category was determined according to a predefined hierarchical rule. Priority was first given to the model that best matched the stated research objective. When the objective was not sufficiently explicit, the model that generated the principal findings or supported the main conclusion was selected. When multiple models were used and the primary model could not be readily distinguished, the study was preferentially assigned to the model category closest to clinical translation. Inter-rater agreement was assessed using percent agreement and Cohen’s kappa before consensus discussion. Disagreements were resolved through discussion, and the final consensus classification was used for analyses.

## 3 Results

### 3.1 Global production and research trends

**Figure 2:**
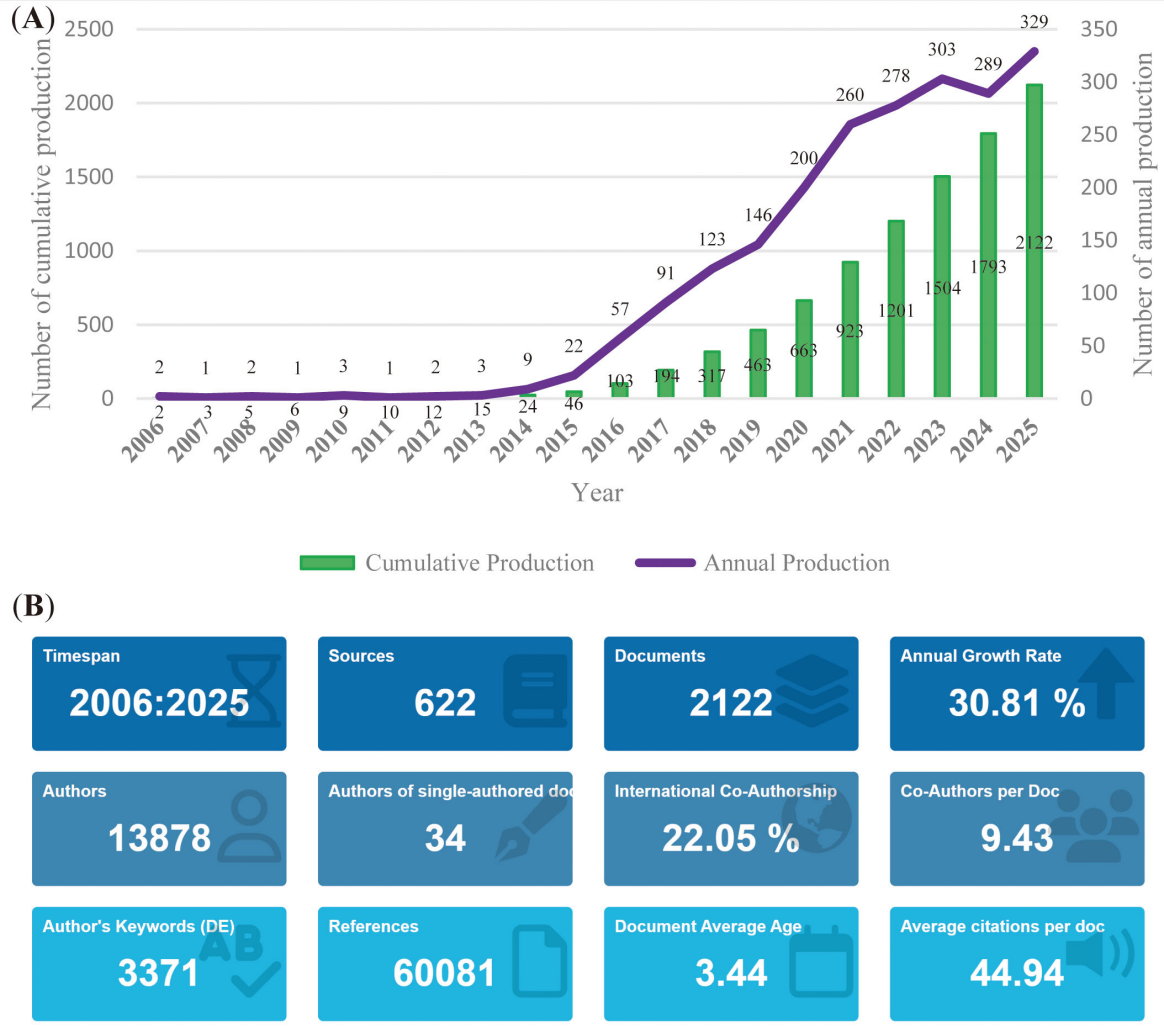
Global production in the field of resistance to PD-1/PD-L1 inhibitors. **(A)** Trends in annual and cumulative publications from 2006 to 2025. **(B)** Additional data regarding global production from the R package Bibliometrix.

We screened 2,122 articles on resistance to PD-1/PD-L1 inhibitors from January 1, 2006, to November 29, 2025. **Figure 2A** illustrates that the cumulative number of publications has increased year by year, indicating that this field is attracting growing attention from researchers. Annual publication counts showed sustained growth in global research output, despite a modest decline in 2024. The growth curve showed two distinct increases in 2014 and 2019. These temporal changes may be interpreted in relation to major regulatory and clinical milestones, such as the FDA’s approval of PD-1 inhibitors for metastatic melanoma in 2014 (27) and the subsequent market approval of China’s first PD-1 inhibitor, toripalimab, in December 2018 (28). Nevertheless, the temporal concurrence does not establish a direct causal relationship. As noted in **Figure 2B**, the compound annual growth rate is 30.81%, indicating strong development momentum in this field. Additional information on sources, authors, and references provides a comprehensive overview of the included literature, offering researchers a deeper understanding of the field.

### 3.2 Distribution and collaboration of countries

**Figure 3:**
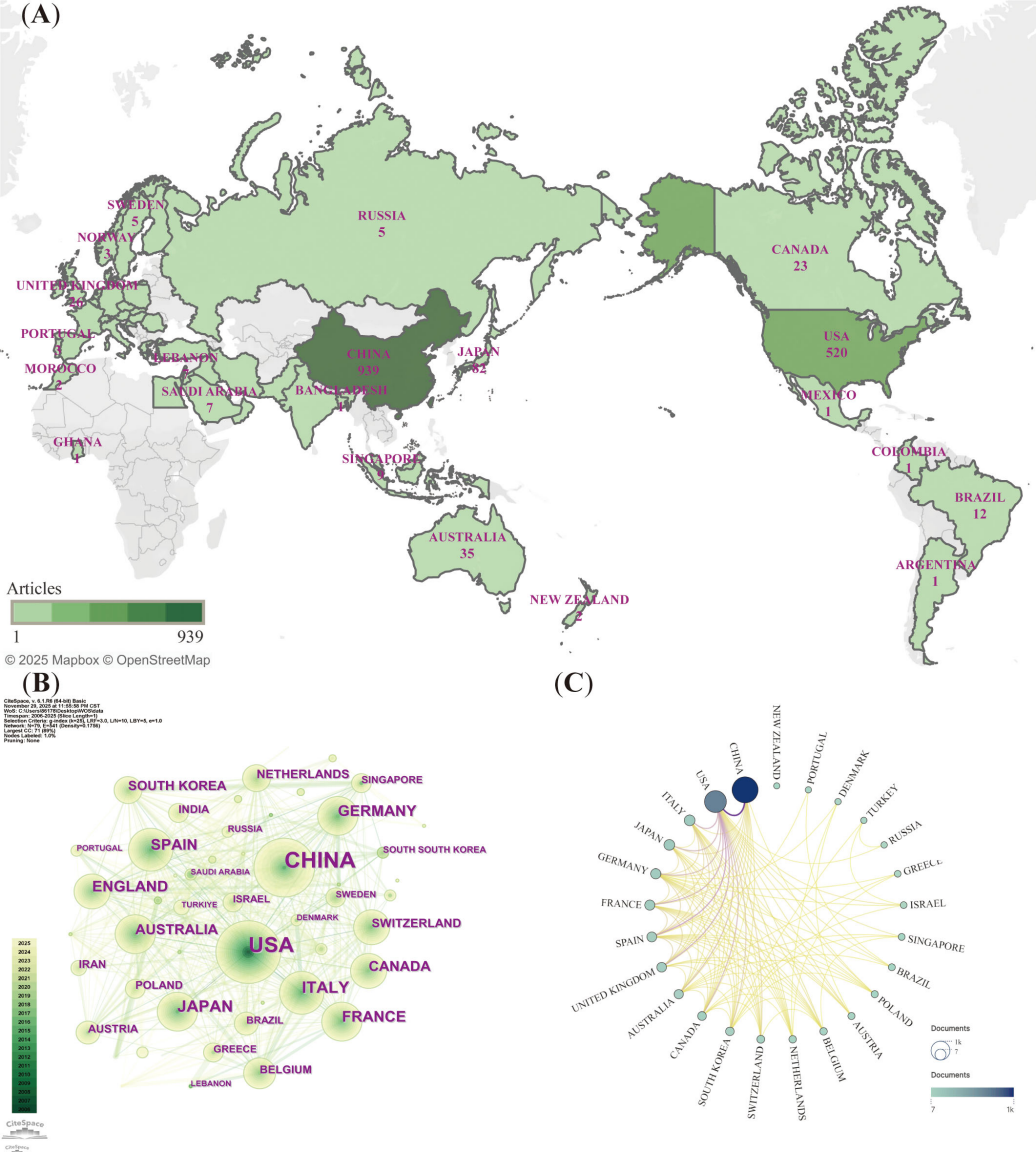
Analysis of countries’ distribution and collaboration. **(A)** Global distribution of countries, with color depth representing the number of publications. **(B)** Network diagram of cooperation among 79 countries by Citespace 6.1.R6. The parameter settings are as follows: timespan: 2006-2025 (slice length=1); g-index (k=25), e=1.0; network: N = 79, E = 541 (Density=0.1756). **(C)** Chord diagram of the cooperation relationship of the top 25 countries with the most significant number of documents by Scimago Graphica 1.0.53. The parameter settings are as follows: size=6; border=1; color weight: number of publications; layout: circular.

We discovered that 79 countries have contributed to the research on PD-1/PD-L1 inhibitor resistance. **Figure 3A** shows a pronounced geographical distribution of global publication output. Asia, Europe, North America, and Oceania are the major contributing regions, while African countries tend to lack research in this field. This pattern reflects an uneven distribution of scientific and medical resources worldwide. It identifies China (n = 939) and the United States (n = 520) as the leading contributors, followed by Japan (n = 82), Italy (n = 75), Germany (n = 57), France (n = 46), Australia (n = 35), Korea (n = 35), Spain (n = 32), and the United Kingdom (n = 26). **Figure 3B** illustrates the cooperation network among countries, with China and the United States being the central countries in the field. Analysis of collaboration relationships among the top 25 productive countries shows a robust bilateral partnership between China and the United States, with the latter maintaining broader multinational ties (**Figure 3C**). As shown in **Table 1**, the United States ranks first in the number of multiple-country publications (MCP = 149), highlighting its pivotal role in international cooperation. Moving forward, it is essential to prioritize the strengthening of global collaborative efforts.

**Table 1:** The top 10 countries with the highest publication counts.

Ranking	Institution	Articles	Percentage	SCP	MCP	MCP %
1	China	939	44.3%	847	92	9.8
2	USA	520	24.5%	371	149	28.7
3	Japan	82	3.9%	64	18	22.0
4	Italy	75	3.5%	48	27	36.0
5	Germany	57	2.7%	39	18	31.6
6	France	46	2.2%	29	17	37.0
7	Australia	35	1.6%	19	16	45.7
8	Korea	35	1.6%	27	8	22.9
9	Spain	32	1.5%	14	18	56.3
10	United Kingdom	26	1.2%	13	13	50.0

MCP, Multiple country publication; SCP, Single country publication.

### 3.3 Comprehensive analysis of institutions

**Figure 4:**
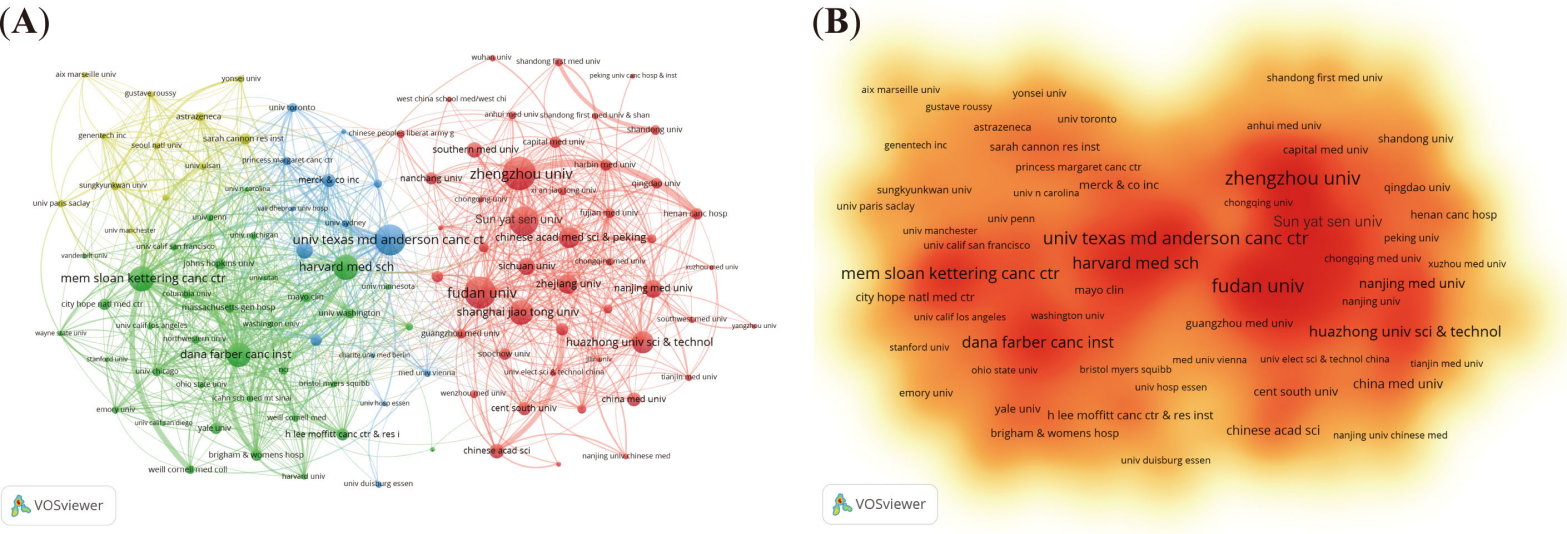
Collaboration map of institutions in the research field of resistance to PD-1/PD-L1 inhibitors by VOSviewer 1.6.20. The parameter settings are as follows: scale=1.15; label size variation= 0.72; cluster resolution=1.0; layout: attraction=3, repulsion=-3; minimum publication count=10; number of nodes=110. **(A)** The collaboration network institutions. Each node in the graphic corresponds to an institution. Its size represents the institution’s documents, and its color indicates the authors’ group. Line thickness is relevant to the strength of collaboration. **(B)** A density visualization of institutions. The darker the color, the more central the institution is in the field.

We discovered that 1580 institutions have participated in the research. **Figure 4A** illustrates the relationships between institutions. Currently, institutional cooperation is primarily domestic, indicating the need for stronger international collaboration. **Figure 4B** highlights the outstanding research contributions and scientific output of various institutions. In conjunction with the data presented in **Table 2**, Zhengzhou University ranks first in publication volume (n = 62), followed by Fudan University (n = 59), with the University of Texas MD Anderson Cancer Center ranking second and third, respectively (n = 57). These institutions demonstrate exceptional research output, significantly advancing the study of PD-1/PD-L1 inhibitor resistance. Additionally, Memorial Sloan Kettering Cancer Center leads in citations (12, 348), followed by the University of Texas MD Anderson Cancer Center (6, 684) and Dana-Farber Cancer Institute (5, 650), underscoring their significant centrality and impact within the field.

**Table 2:** The top 10 institutions with the highest publication numbers.

Ranking	Institution	Articles	Citations
1	Zhengzhou University	62	2870
2	Fudan University	59	1390
3	UTMD Anderson Cancer Center	57	6684
4	Sun Yat Sen University	55	1950
5	Memorial Sloan Kettering Cancer Center	47	12348
6	Harvard Medical School	47	3282
7	Dana-Farber Cancer Institute	46	5650
8	Shanghai Jiao Tong University	46	1709
9	Huazhong University of Science and Technology	41	2378
10	Chinese Academy of Medical Sciences – Peking Union Medical College	40	990

### 3.4 Citation and co-citation of journals

**Figure 5:**
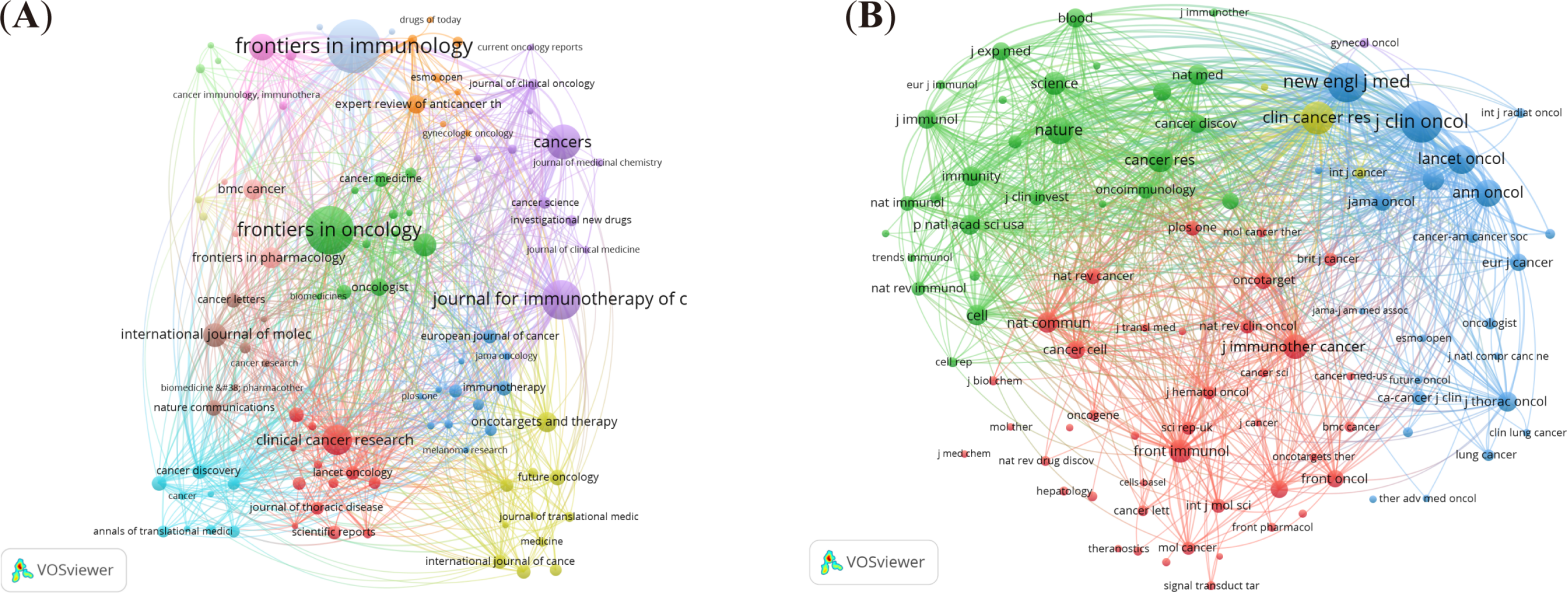
Analysis of journals and co-cited journals in the field of resistance to PD-1/PD-L1 inhibitors by VOSviewer 1.6.20. The parameter settings are as follows: scale=1.05; label size variation= 0.50; cluster resolution=1.0; layout: by Pajek 6.01. The node size encodes the journal’s citations or documents, and the node color represents the journal’s scholarly classification. Line thickness is relevant to the strength of co-citation, or the relationship between journals. **(A)** A collaboration network of 99 journals with a minimum publication count of 5 documents. **(B)** A co-citation network of 151 journals with a minimum of 100 citations.

Global publications were published in 622 journals. According to the collaboration analysis of journals in **Figure 5A**, *Frontiers in Immunology*, *Frontiers in Oncology*, *Journal for Immunotherapy of Cancer*, *Cancers*, and *Clinical Cancer Research* have high publication output on PD-1/PD-L1 inhibitor resistance. These journals play an important role in disseminating academic results and enhancing opportunities for academic communication. As shown in **Table 3** and **Figure 5B**, the top 10 journals by citations are all ranked in Q1, reflecting their high academic influence and wide recognition. Among them, *Journal of Clinical Oncology* (IF = 43.4) ranked first, followed by *The New England Journal of Medicine* (IF = 78.5), *Clinical Cancer Research* (IF = 10.2), *Lancet Oncology* (IF = 35.9), and *Annals of Oncology* (IF = 65.4). These journals play a key role in addressing PD-1/PD-L1 inhibitor resistance. Citation counts do not always correlate directly with the impact factor. Compared to *The New England Journal of Medicine*, *the Journal of Clinical Oncology has a slightly lower impact factor but the highest citation count, indicating its specialization and authority in* oncology. Furthermore, oncology-focused journals such as *Lancet Oncology*, *Annals of Oncology*, and *Clinical Cancer Research* also made significant contributions to the field.

**Table 3:** The top 10 journals with the highest citations.

Ranking	Journal	Citations	IF	JCR quartile
1	Journal of Clinical Oncology	5018	43.4	Q1
2	The New England Journal of Medicine	4357	78.5	Q1
3	Clinical Cancer Research	3116	10.2	Q1
4	Lancet Oncology	2376	35.9	Q1
5	Annals of Oncology	2114	65.4	Q1
6	Nature	2082	48.5	Q1
7	Cancer Research	1859	16.6	Q1
8	Journal for ImmunoTherapy of Cancer	1736	10.6	Q1
9	Frontiers in Immunology	1508	5.9	Q1
10	Science	1471	45.8	Q1

IF, Impact factor; JCR, Journal Citation Reports.

The data was retrieved from Journal Citation Reports (JCR) in 2024.

### 3.5 Authors and co-cited authors

**Figure 6:**
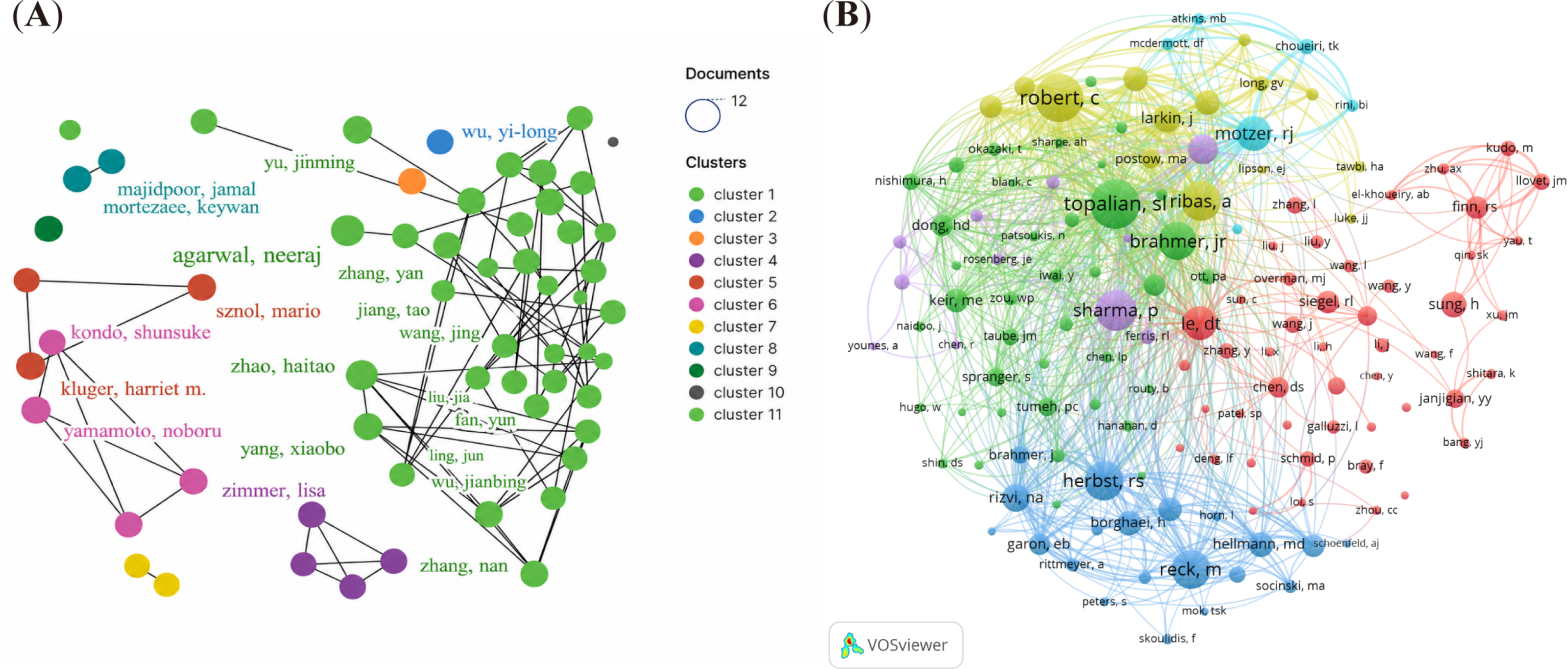
Analysis of authors and co-cited authors in the research field of resistance to PD-1/PD-L1 inhibitors. The parameter settings are as follows: VOSviewer: scale=1.05; label size variation= 0.50; cluster resolution=1.0; layout: attraction=3, repulsion=0; minimum citations=50; number of nodes=151. Scimago Graphica: size=5; border=1; minimum counts=6; color weight: Number of publications; layout: force directed. The node size encodes the author’s citations or documents, and the node color represents their scholarly classification. Line thickness is relevant to the strength of co-citation, or the relationship between authors. **(A)** A collaboration network of 58 authors by Scimago Graphica 1.0.53. **(B)** A co-citation network of authors by VOSviewer 1.6.20.

We retrieved 13878 authors who have contributed to this field. **Figure 6A** visualizes the contributions and collaboration network of authors, with color clusters representing their scholarly classification. The results indicate a relatively high proportion of Chinese authors. Furthermore, authors tended to collaborate more frequently within their own countries, while international collaboration was comparatively limited. The ten most productive authors were listed in **Table 4**. Among them, Neeraj Agarwal is the leading author, ranking first in the number of published documents (n = 10). His research primarily focuses on overcoming PD-1/PD-L1 inhibitor resistance in urothelial carcinoma by combining these inhibitors with other therapeutic modalities, such as targeted therapies (29).

**Table 4:** The top 10 productive authors.

Ranking	Author	Articles	Institution
1	Agarwal, Neeraj	10	Univ Utah
2	Zhao, Haitao	9	Chinese Acad Med Sci, Peking Union Med Coll Hosp
2	Yang, Xiaobo	8	Chinese Acad Med Sci, Peking Union Med Coll Hosp
4	Zimmer, Lisa	8	Univ Hosp Essen
4	Koyama, Takafumi	8	Natl Canc Ctr
4	Yamamoto, Noboru	8	Natl Canc Ctr
4	Kluger, Harriet M.	8	Yale Univ
4	Yang, Xu	8	Chinese Acad Med Sci, Peking Union Med Coll Hosp
9	Zhang, Nan	7	Chinese Acad Med Sci & Peking Union Med Coll CAMS, Peking Union Med Coll Hosp
9	Shimizu, Toshio	7	Natl Canc Ctr, Dept Expt Therapeut

**Figure 6B** highlights collectively influential authors and the scientific community, as visualized by VOSviewer. Researchers such as Stephen L. Topalian, Antoni Ribas, and Padmanee Sharma play a central role in the research network on resistance to PD-1/PD-L1 inhibitors. Among them, Stephen L. Topalian was the most central author (according to the citation counts in **Supplementary Table 3**), a pioneer in the clinical application of PD-1/PD-L1 immune checkpoint inhibitors (30). In 2014, Stephen et al. conducted a systematic analysis of the tumor microenvironment in patients treated with the anti-PD-1 agent nivolumab. The study was the first to demonstrate that PD-L1 protein expression on tumor cells is the most critical biomarker for predicting clinical benefit from anti-PD-1 immunotherapy (31).

### 3.6 Analysis of reference

A total of 60,081 references were identified in this field. To understand the critical research in this field, we identified the top 10 references by cited counts using the R package Bibliometrix. The titles, first authors, and DOI of these references are listed in **Supplementary Table 4**. The top 3 references with high citations were led by BARBER DL (2006, NATURE) (total citations = 3437), POSTOW MA (2015, J CLIN) (total citations = 2209), and GORDON SR (2017, NATURE ONCOL) (total citations = 1805). Barber et al. (2006) reported that virus-specific CD8^+^ T cells in an exhausted state exhibited high levels of PD-1 expression. Importantly, blockade of the PD-1/PD-L1 interaction reversed T-cell exhaustion and restored T-cell function. These findings suggest that the PD-1/PD-L1 pathway represents a key mechanism underlying T-cell dysfunction and tumor immune evasion (32). Postow et al. (2015) systematically reviewed early clinical progress and prospects of immune checkpoint inhibitors targeting PD-1/PD-L1 in multiple cancers (33). Gordon et al. (2017) revealed that tumor-associated macrophages (TAMs) also express PD-1. Blockade of the PD-1/PD-L1 axis enhanced TAM-mediated phagocytosis and suppressed tumor growth. The authors further suggested that PD-1 signaling in immunosuppressive myeloid cells within the tumor microenvironment, including M2-polarized TAMs, may attenuate therapeutic efficacy. Therefore, targeting this pathway may help overcome resistance by activating multiple immune cell populations (34).

**Figure 7:**
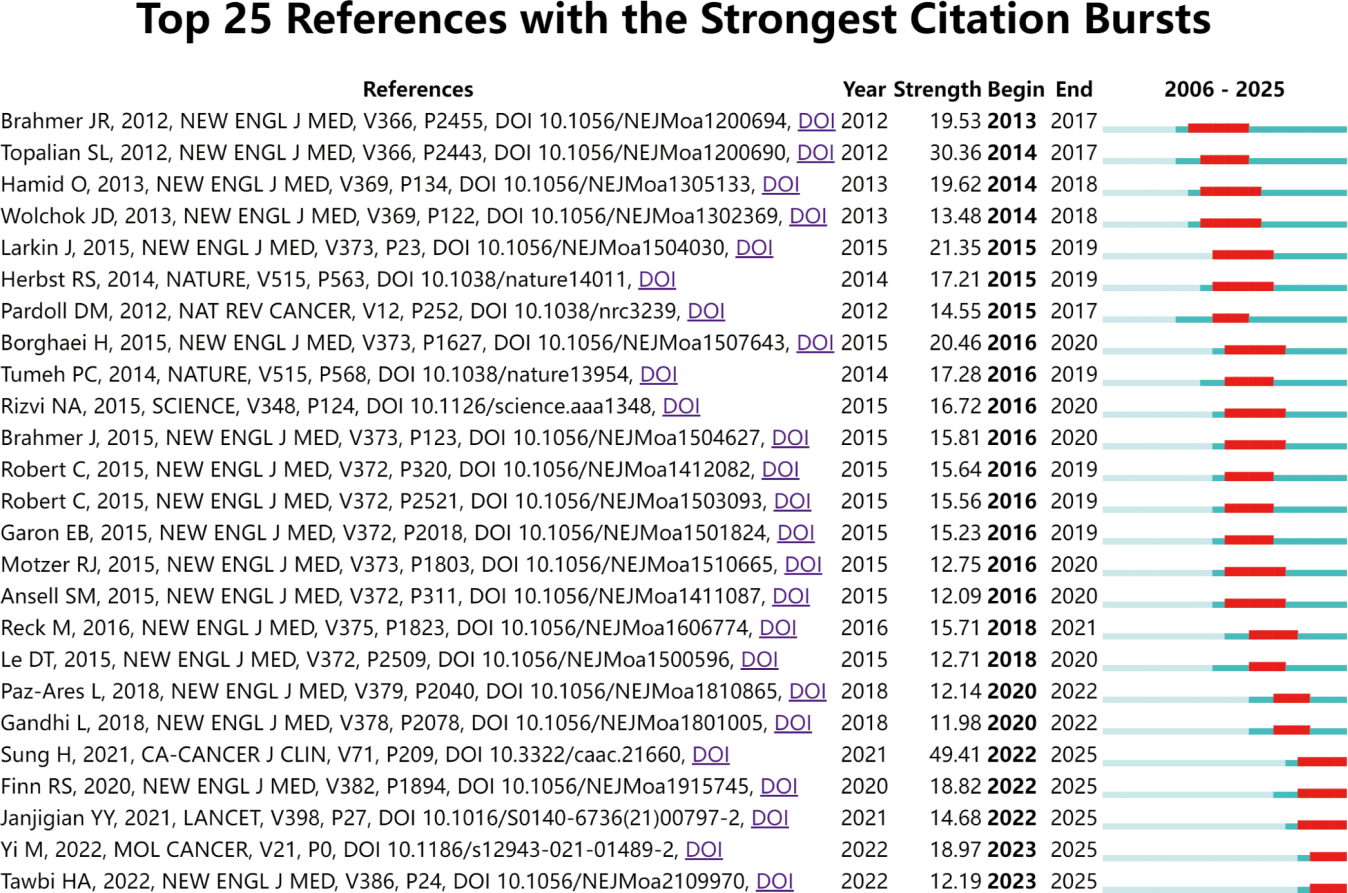
The top 25 references with a significant increase in citation frequency by Citespace. The blue timeline illustrates the dates of the bursts, the start and end of each burst, and the time intervals between them.

**Figure 7** illustrates the top 25 references with the highest burst strength. By analyzing citation counts, high-impact studies at different time points can be identified, thereby reflecting the evolution of research on PD-1/PD-L1 inhibitor resistance. Citation bursts in references over the past two years may highlight current research hotspots and emerging future trends. For example, Yi et al. comprehensively reviewed recent clinical advances in combination therapies based on PD-1/PD-L1 inhibitors, particularly from a molecular and immunological perspective. They further highlighted potential future research directions, including novel bispecific antibodies and personalized combination approaches (35). In a phase III clinical trial, Tawbi et al. showed that the combination of the Lymphocyte activation gene-3 (LAG-3) inhibitor relatlimab with the PD-1 inhibitor nivolumab significantly prolonged progression-free survival, thereby helping address the efficacy limitations of nivolumab monotherapy due to resistance (36). These two studies consistently highlighted the critical role and translational potential of combination strategies in overcoming resistance to PD-1/PD-L1 inhibitors.

### 3.7 Keyword visualization and burst test

Our study identified 9,603 keywords from all documents. To explore specific research hotspots and future trends, we analyzed keyword co-occurrences using VOSviewer and the R package Bibliometrix. Additionally, we conducted a citation burst test and created a keyword timeline visualization using Citespace.

**Figure 8:**
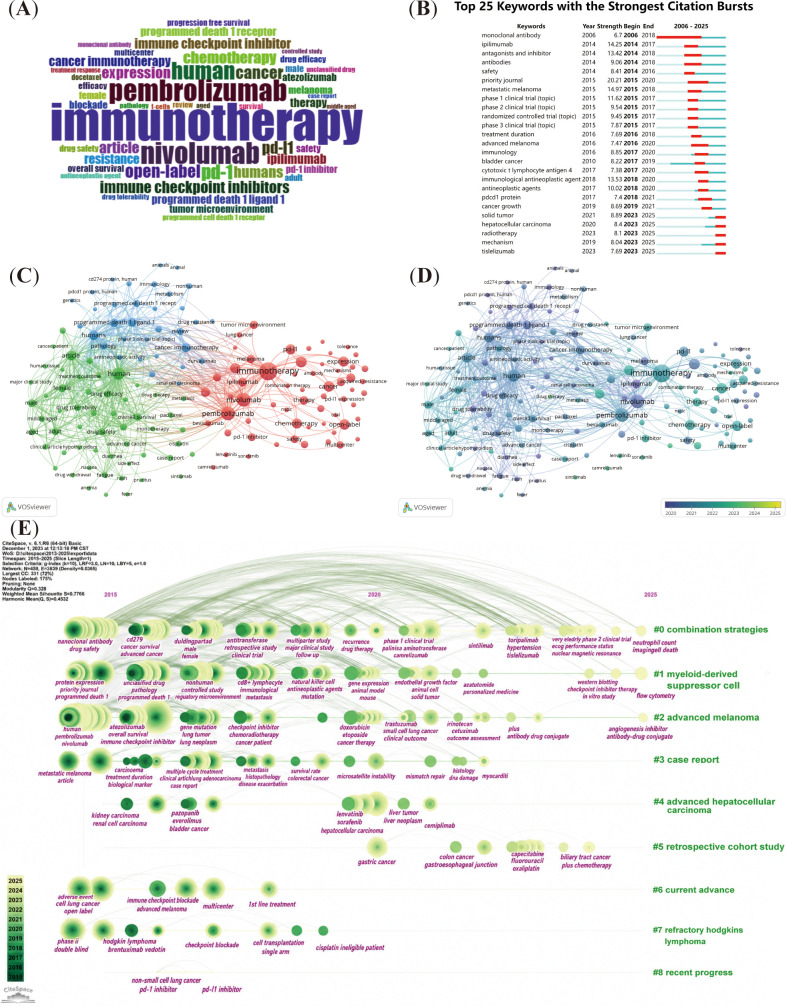
Analysis of keywords in resistance to PD-1/PD-L1 inhibitors. The parameter settings are as follows: R package Bibliometrix: word occurrence=frequency; shape=circle; font type=impact; font size=1.0; ellipticity=0.65; padding=1; rotate=0. VOSviewer: scale=1.05; label size variation= 0.50; cluster resolution=1.0; layout: attraction=3, repulsion=0; minimum citations=50; number of nodes=148. Citespace: timespan=2015-2025 (slice length=1); g-index (k=10), e=1.0. cluster methods: log-likelihood ratio (LLR). **(A)** The word cloud of keywords: highlight central keywords in this field by R package Bibliometrix 4.4.2. **(B)** The top 25 keywords with the most powerful citation bursts by Citespace 6.1.R6. **(C)** Visualization of the co-occurrence network of keywords by VOSviewer 1.6.20. Each node in the network corresponds to a specific keyword. The links represent keyword co-occurrence, and the frequency of a term is defined by node size. **(D)** Visualization of network analysis of keywords in terms of time. The links represent keyword co-occurrence, and the size of the node defines the frequency of a term. The lighter the color, the later it appears by VOSviewer 1.6.20. **(E)** Timeline of keywords visualization by Citespace 6.1.R6.

As shown in **Supplementary Table 5** and **Figure 8A**, the co-occurrence data indicated that “immunotherapy” (occurrence = 844) and “cancer” (occurrence = 275) underscore the important role of immunotherapy in cancer treatment. “Pembrolizumab” (occurrence = 492) and “nivolumab” (occurrence = 486) are first-line immunotherapeutic agents for multiple cancer types and have also become a major focus of resistance research. “Humans” (occurrence = 397) and “open-label” (occurrence = 301) reflected the important role of clinical trials in investigating resistance to PD-1/PD-L1 inhibitors. “PD-1” (occurrence = 324) and “PD-L1” (occurrence = 279) highlighted the emphasis on mechanistic pathways and biomarkers in resistance research. “Chemotherapy” (occurrence = 270) suggested a combination strategy for overcoming resistance to PD-1/PD-L1 inhibitors. **Figure 8B** presents the top 25 keywords with the strongest citation bursts. Burst detection identifies periods of sharply increased keyword frequency, highlighting emerging research hotspots. In the past two years, burst keywords such as “solid tumor,” “hepatocellular carcinoma,” “radiotherapy,” “mechanism,” and “tislelizumab” reflect the growing attention on PD-1/PD-L1 inhibitor resistance in solid tumors. They also indicate that the development of novel drugs has become a current research focus. **Figures 8C, D** shows the co-occurrence network and time sequence of keywords by VOSviewer. Keywords such as “tumor microenvironment” and “mechanisms” indicate that research on drug resistance mechanisms is no longer focused solely on tumor cells, but has expanded to the entire tumor microenvironment (37).

**Figure 8E** shows the keyword timeline visualization, which highlights the chronological development of key research themes. The time span was set from 2015 to 2025, with 1 year per slice. The node selection criterion was g-index (k = 20), and no pruning was applied. Different clusters represent distinct research themes. Cluster #0, “combination strategies,” was the largest cluster, indicating that overcoming PD-1/PD-L1 inhibitor resistance through combination therapy has remained a central theme in this field. Its early keywords were mainly concentrated on “monoclonal antibody,” “drug safety,” “advanced cancer,” and “cancer survival,” suggesting that initial research primarily focused on evaluating the safety and preliminary efficacy of PD-1/PD-L1 monoclonal antibodies in patients with advanced malignancies. This theme subsequently evolved toward terms such as “retrospective study,” “clinical trial,” and “multicenter study,” reflecting a shift from single-agent clinical observation to the accumulation of multicenter evidence and the optimization of combination strategies. After 2020, the emergence of keywords such as “sintilimab,” “toripalimab,” “tislelizumab,” “ECOG performance status,” and “neutrophil count” suggests that the field expanded into the iterative development of PD-1/PD-L1 agents, patient stratification in special populations, and the refined management of combination regimens. Cluster #1, “myeloid-derived suppressor cell,” included keywords such as “tumor microenvironment,” “CD8+ T lymphocyte,” “gene expression,” and “*in vitro* study,” indicating that research on resistance mechanisms has clearly shifted from a tumor cell-centered perspective toward the broader immunosuppressive microenvironment and the interactions among multiple immune cell populations. In this context, myeloid-derived suppressor cells, T-cell dysfunction, and related alterations in molecular expression have emerged as key areas for mechanistic investigation.

The remaining clusters further illustrate the continued extension of this field into specific tumor types and precision therapeutic settings. In cluster #2, “advanced melanoma,” the progression of keywords from “pembrolizumab” and “nivolumab” to “chemoradiotherapy” and “angiogenesis inhibitor” suggests that immunotherapy for advanced melanoma has evolved from a focus on PD-1/PD-L1 monotherapy toward combination approaches involving chemoradiotherapy, antibody-drug conjugates, and anti-angiogenic therapy to address resistance. Clusters #4, “advanced hepatocellular carcinoma,” and #7, “refractory Hodgkin lymphoma,” indicate that resistance to PD-1/PD-L1 inhibitors poses a challenge across both solid and hematologic malignancies, underscoring the importance of investigating tumor-specific mechanisms of resistance. In cluster #3, “case report,” keywords such as “biological marker,” “microsatellite instability,” “mismatch repair,” and “DNA damage” highlight the growing importance of biomarkers in cancer treatment. These findings highlight the potential value of biomarker-based patient selection. Such an approach may help define patient populations that are more likely to benefit from PD-1/PD-L1 inhibitors.

### 3.8 Research classification based on experimental models

A total of 1,466 studies were included in the experimental model classification. The initial agreement between the two reviewers was 84.5%, with a Cohen’s kappa of 0.796, indicating substantial agreement. The final model-classification dataset is listed in **Supplementary Table 6**. As shown in **Table 5**, the literature was predominantly based on human clinical samples (n = 1,198, 81.7%), followed by mouse models (n = 197, 13.4%), *in vitro* studies (n = 66, 4.5%), other animal models (n = 3, 0.2%), and organoid studies (n = 2, 0.1%). Notably, the near absence of organoid studies suggests a lack of an intermediate platform between *in vitro* experiments and clinical studies. This may limit mechanistic validation and treatment-response prediction, and in turn reduce the efficiency and success of RCTs design. Within the human clinical sample category, study designs were unevenly distributed: retrospective studies were the most common (n = 631, 52.7%), followed by prospective studies (n = 297, 24.8%) and case series (n = 189, 15.8%), whereas RCTs accounted for only a small proportion (n = 81, 6.8%). More intervention verifications are needed to promote clinical translation and strategic research in the future.

**Table 5:** Research classification based on experimental models.

Experimental model	Articles	Percentage
Human Clinical Samples(RCTs)	81	6.8%
Human Clinical Samples(Prospective study)	297	24.8%
Human Clinical Samples(Retrospective study)	631	52.7%
Human Clinical Samples(Case series)	189	15.8%
Organoids	2	0.1%
Mouse Models	197	13.4%
Other Animal Models	3	0.2%
*In Vitro* Studies	66	4.5%

RCTs, Randomized controlled trials.

## 4 Discussion

### 4.1 Global contribution and academic background

Research on PD-1/PD-L1 inhibitor resistance has expanded significantly in recent years, particularly in terms of its mechanisms and therapeutic strategies (38). China is dominant in research output. This trend may be attributed to China’s large population and substantial cancer burden. In 2022, China reported approximately 4.82 million new cancer cases and 2.57 million cancer-related deaths, with the burden continuing to rise (39). In addition, national initiatives such as “Healthy China 2030” have promoted early cancer screening, standardized diagnosis and treatment, and expanded clinical research. These efforts have also stimulated increased investment in research on mechanisms of immunotherapy resistance (40). Among PD-1/PD-L1-targeted therapies, tislelizumab is a domestically developed PD-1 inhibitor approved in China in 2019 and now approved in multiple international markets. Compared with conventional PD-1 antibodies, a key feature of tislelizumab is its engineered Fc region, which reduces its binding affinity for Fcγ receptors. This modification may decrease the likelihood of macrophage-mediated phagocytosis of effector T cells in the tumor microenvironment, thereby helping to reduce the development of therapeutic resistance (41). The United States has demonstrated outstanding achievements in both scientific research output and international collaboration. It was among the first to approve PD-1/PD-L1 inhibitors, including pembrolizumab, nivolumab, atezolizumab, and durvalumab, which helped shift cancer treatment from conventional chemotherapy to immunotherapy (42). In addition, the United States was among the first to propose and implement a tumor-agnostic immunotherapy model guided by biomarkers such as Microsatellite Instability-High (MSI-H) or deficient Mismatch Repair (dMMR) and Tumor Mutational Burden-High (TMB-H). This development has promoted a transition in anticancer therapy from treatment decisions based solely on tumor site to precision treatment guided by molecular features, helping reduce the risk of therapeutic resistance (43). In addition, European countries, as well as Japan and Australia, have also made notable contributions. Future efforts to strengthen international collaboration may further accelerate progress in research on therapeutic resistance.

At the institutional level, Memorial Sloan Kettering Cancer Center has coordinated multiple key clinical trials and systematic data collection on PD-1/PD-L1 therapies. These efforts have provided a structured dataset and rigorous methodologies for studying mechanisms of therapeutic resistance. Journals such as the *Journal of Clinical Oncology* and *The New England Journal of Medicine* are at the forefront of high-impact research in this field. They offer platforms for academic exchange and serve as practical references for researchers and clinicians worldwide. Neeraj Agarwal and Stephen L. Topalian are the most prolific and the most influential authors, respectively. Through high-quality studies, they have elucidated mechanisms of PD-1/PD-L1 inhibitor resistance and assessed potential combination therapeutic strategies. Looking forward, broad collaboration among institutions and scholars, coupled with multicenter prospective clinical trials, will be critical to generating robust clinical evidence and addressing therapeutic resistance.

### 4.2 Description of research hotspots and future trends

**Figure 9:**
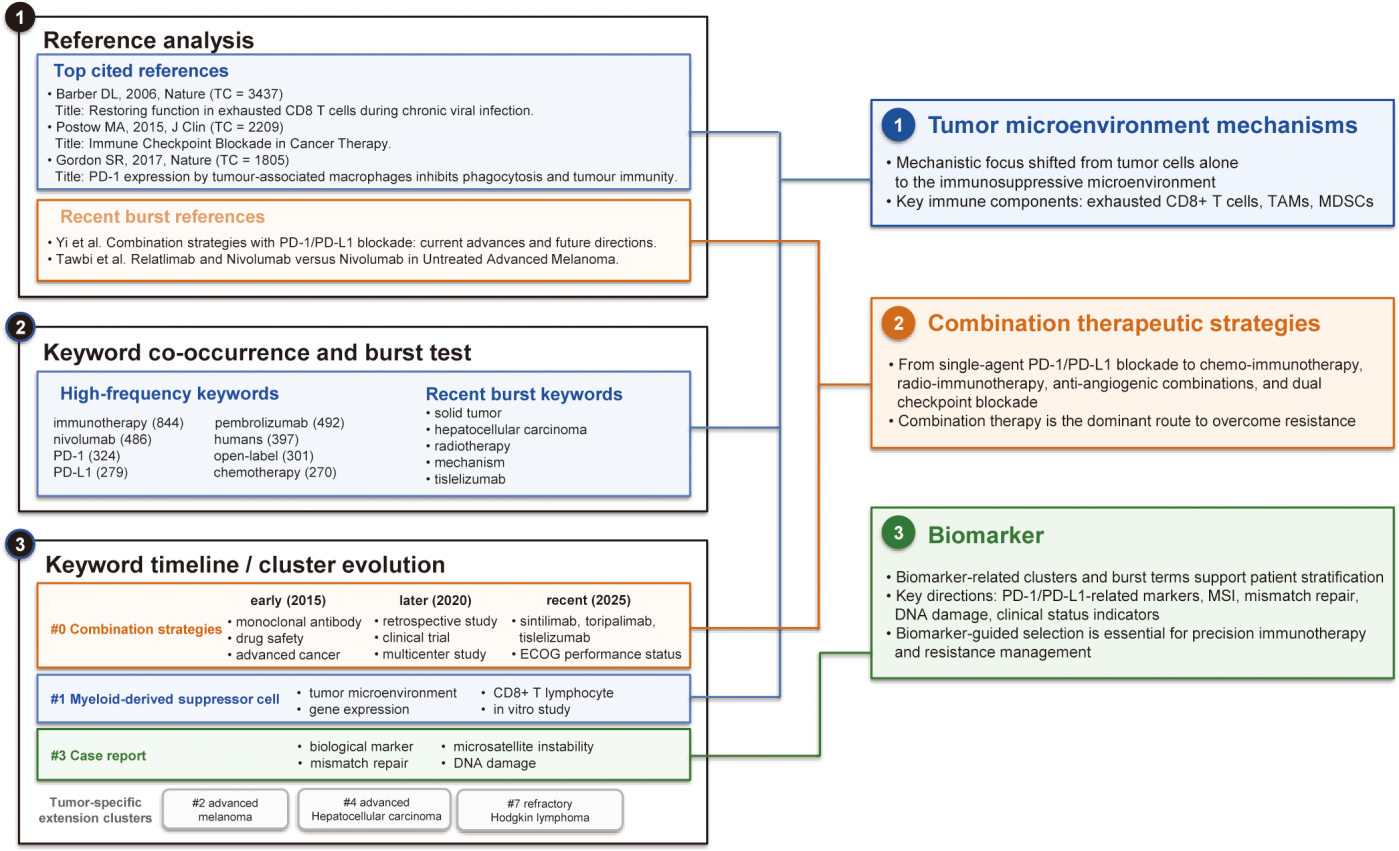
A summary framework linking reference analysis, keyword analysis, burst detection, and timeline clusters to the study conclusions.

Based on an analysis of highly cited references, emerging citations, co-occurring keywords, keyword bursts, and keyword timeline mapping, we have identified key research hotspots and trends in PD-1/PD-L1 inhibitor resistance. As shown in **Figure 9**, these findings can be summarized into four main points: (1) Resistance mechanisms in the tumor microenvironment; (2) Overcoming resistance and enhancing efficacy through combination therapies. (3) The prediction of treatment response by biomarkers; (4) More RCTs and organoid research are needed to promote clinical translation in the future. In the following part, we will examine each of these four aspects in detail.

#### 4.2.1 Mechanism of PD-1/PD-L1 inhibitor resistance in the tumor microenvironment

Resistance is not solely due to tumor-cell adaptation. It is also shaped by the tumor immune microenvironment (TME). The TME includes immune cells, stromal cells, and signaling molecules. It supports tumor growth and promotes resistance through several key mechanisms (44). Common mechanisms include defective antigen presentation, impaired interferon signaling, and progressive T-cell dysfunction (45). Immunosuppressive cells are recruited into the TME, including regulatory T cells (Tregs), myeloid-derived suppressor cells (MDSCs), and TAMs. They secrete factors such as Transforming Growth Factor-beta (TGF-β) and interleukin-10 (IL-10) to suppress effector T cells and weaken the immune response to PD-1/PD-L1 inhibitors (46). Metabolic changes also drive resistance to PD-1/PD-L1 inhibitors. Tumors enhance glycolysis and tryptophan metabolism to produce metabolites like kynurenine and adenosine. These compounds directly inhibit T cell function and promote exhaustion (47). In addition, intratumoral heterogeneity creates diversity among cancer cells. Different subclones express varying antigens and signaling states. This variability influences immune infiltration and the response to PD-1/PD-L1 inhibitors (48).

Resistance mechanisms to PD-1/PD-L1 inhibitors share commonalities across tumor types, but also exhibit significant heterogeneity (49). In non-small cell lung cancer (NSCLC), the Kirsten rat sarcoma viral oncogene homolog, G12D (KRAS-G12D) mutation downregulates the High mobility group AT-hook 2–C-X-C motif chemokine ligand 10/C-X-C motif chemokine ligand 11 (HMGA2-CXCL10/CXCL11) axis. It inhibits CD8+ T cell recruitment and is associated with low PD-L1 expression, thereby driving primary resistance (50). Additionally, Trop2 overexpression is closely linked to T cell exclusion and immunotherapy tolerance in NSCLC (51). In small cell lung cancer (SCLC), lineage plasticity, characterized by transitions among the Achaete-Scute Family BHLH Transcription Factor 1 (ASCL1), Neurogenic Differentiation 1 (NEUROD1), POU Class 2 Homeobox 3 (POU2F3), and Yes-Associated Protein 1 (YAP1) molecular subtypes, constitutes a critical mechanism of acquired resistance (52). In melanoma, tyrosinase can mediate resistance by maintaining an immunologically “cold” state (53). Ambra1 can promote the IL-1α-C-C motif ligand 22 (CCL22) axis to recruit immunosuppressive Tregs into the TME, thereby driving resistance to PD-1/PD-L1 inhibitors (54). In renal cell carcinoma (RCC), particularly the clear cell subtype (ccRCC), Secreted Phosphoprotein 1 (SPP1) + TAMs promote resistance to PD-1/PD-L1 inhibitors by directly inducing CD8+ T cell dysfunction (55). The emerging immune checkpoint Variable Immunoglobulin Superfamily Member 4 (VSIG4) also shapes an immunosuppressive microenvironment in RCC by recruiting M2 macrophages and Tregs, thereby facilitating resistance to PD-1/PD-L1 inhibitors (56). In urothelial carcinoma (UC), the highly expressed cytokine Growth Differentiation Factor 15 (GDF-15) inhibits the Lymphocyte Function-Associated Antigen 1 (LFA-1)/Intercellular Adhesion Molecule 1 (ICAM-1) axis to impede T cell infiltration. This mediates immune evasion and PD-1/PD-L1 inhibitor resistance, a mechanism also observed in NSCLC (57). In colorectal cancer (CRC), microsatellite stable (MSS) tumors are refractory to PD-1/PD-L1 inhibitors due to their low immunogenicity and an immunosuppressive TME (58). Classical Hodgkin lymphoma (cHL) overexpresses PD-L1 due to its characteristic 9p24.1 amplification, rendering it highly responsive to PD-1 blockade. However, resistance can still emerge, predominantly driven by T-cell depletion (59).

Currently, elucidating drug resistance heavily relies on retrospective analyses. Moving forward, the integration of single-cell sequencing and spatial transcriptomics promises to proactively map these resistance architectures, unveiling timely targets for precise intervention (60).

#### 4.2.2 Combination strategy to overcome the resistance of PD-1/PD-L1 inhibitors

Combination therapy is a major strategy for overcoming resistance to PD-1/PD-L1 inhibitors. Monotherapy is often limited by primary or acquired resistance (61). For this reason, current clinical practice increasingly relies on rational combinations that enhance T-cell priming, improve immune-cell infiltration, or reverse immunosuppressive signaling (62). Among these approaches, combinations with chemotherapy, radiotherapy, anti-angiogenic agents, and other immune checkpoint inhibitors have shown the greatest translational and clinical relevance (11, 63–65).

Researchers are actively exploring combination strategies across various tumor types to overcome PD-1/PD-L1 inhibitor resistance. In lung cancer, a phase III trial evaluating a “four-drug regimen” (the anti-angiogenic agent anlotinib and the PD-L1 inhibitor benmelstobart combined with chemotherapy) achieved the longest median overall survival to date in SCLC. It confirms the synergistic potential of incorporating anti-angiogenic drugs to overcome PD-1/PD-L1 inhibitor resistance (66). Additionally, combining the Delta-Like 3 (DLL3)-targeted bispecific T-cell engager tarlatamab with a PD-L1 inhibitor directly activates T cells in an (Major Histocompatibility Complex class I (MHC-I)-independent manner, bypassing the antigen presentation defects common in SCLC (67). In NSCLC, the Mitogen-Activated Protein Kinase Kinase (MEK) inhibitor trametinib remodels the TME by downregulating Inhibitor of DNA binding 1 (Id1), while bezafibrate promotes fatty acid oxidation in T cells; both strategies show promise in sensitizing tumors to PD-1/PD-L1 inhibitors (68). In melanoma, the CD40 agonist sotigalimab combined with nivolumab has induced durable responses in PD-1-resistant patients (69). Furthermore, fecal microbiota transplantation (FMT) transforms “resistant microbiomes” into “responder microbiomes” by remodeling gut microbiota, offering a novel approach to overcoming PD-1/PD-L1 inhibitor resistance (70). For MSS CRC, researchers have developed an oral, gut-microbiota-responsive nanoplatform. This system co-delivers immunogenic cell death inducers and Dendritic Cell (DC) maturation agents to convert “cold” tumors into “hot” ones, thereby improving responsiveness (71). In urological tumors, intravesical instillation of VAX014 combined with PD-L1 blockade induces both local and systemic anti-tumor immunity in bladder cancer (72). The combination of savolitinib and durvalumab has also shown durable efficacy in MET-driven metastatic papillary renal cell carcinoma, effectively ameliorating PD-1/PD-L1 inhibitor resistance (73). In cHL, combining PD-1 inhibitor with the Histone Deacetylase (HDAC) inhibitor vorinostat or the hypomethylating agent decitabine restores tumor sensitivity to immunotherapy via epigenetic regulation (74).

Despite numerous combination therapies being explored, clinical translation remains suboptimal. Overcoming this challenge requires a mechanism-driven approach that leverages robust biomarker profiling to truly realize precision oncology (75).

#### 4.2.3 Predict PD-1/PD-L1 inhibitor resistance by biomarkers

The identification of biomarkers to predict response to PD-1/PD-L1 inhibitors is a promising approach to avoid drug resistance (76). PD-L1 expression, MSI-H, TMB-H, and immune cell infiltration profiles are currently widely used to predict therapy efficacy across various cancer types. Research indicates that patients with these biomarker features respond well to PD-1/PD-L1 inhibitors (77, 78).

As research advances, the types and scope of biomarkers continue to expand. Researchers have identified tumor-specific biomarkers to prevent resistance and achieve highly effective immunotherapy. In NSCLC, high expression of the microRNA (miR)-23a/27a/24–2 cluster can predict the failure of PD-1/PD-L1 inhibitor monotherapy (79). Meanwhile, the recovery of peripheral blood lymphocytes post-chemotherapy serves as a simple, dynamic indicator of potential benefit from subsequent PD-1 blockade (80). In SCLC, circulating exosomal PD-L1 levels can act as a liquid biopsy biomarker to predict efficacy and monitor the onset of resistance (81). For melanoma, patients with lower baseline microbial diversity may more readily overcome resistance and benefit from FMT combined with immunotherapy (82). In CRC, MSI-H/dMMR status remains the most robust predictive biomarker for identifying patients likely to benefit from PD-1/PD-L1 inhibitors (83). In urological tumors, the ratio of PD-1+ Tregs to CD8+ T cells in ccRCC accurately identifies patients less likely to develop resistance (84). In UC, high NECTIN4 expression is a potential biomarker for selecting optimal candidates for combined PD-1 and Nectin-4 targeted antibody drug conjugate (ADC) (85). In cHL, T-cell depletion is driven by the loss of central memory T cells and the upregulation of (T-cell immunoglobulin and mucin-domain containing-3) TIM-3 and LAG-3. Consequently, these inhibitory receptors serve as valuable predictive biomarkers for therapeutic resistance and guiding treatment decisions (86).

In conclusion, future efforts must integrate tumor-specific molecular features, the composition of the immune microenvironment, and circulating factors to construct mechanism-driven biomarker models. This integration is essential to achieve the core goals of precisely screening optimal patient populations and effectively overcoming PD-1/PD-L1 inhibitor resistance (87).

#### 4.2.4 More RCTs and organoid research are needed to promote clinical translation

By analyzing the experimental models and research designs of the included studies, this review attributes the low clinical translation rate in PD-1/PD-L1 inhibitor resistance research to a structurally imbalanced evidence base and a lack of intermediate translational models. Most current research utilizes human samples. However, the evidence relies heavily on observational studies, such as retrospective cohorts and case series, and lacks RCTs to demonstrate intervention efficacy (88). Moreover, traditional *in vitro* cell lines and mouse models offer limited predictive value because they fail to accurately recapitulate the complex human tumor immune microenvironment (89). The notable absence of organoid models further exposes a critical gap in functional validation and drug screening between basic discovery and clinical application (90). Therefore, future efforts should prioritize rigorous RCTs and patient-derived organoid (PDO) studies to accelerate clinical translation and overcome PD-1/PD-L1 inhibitor resistance.

### 4.3 Limitation

Although we have conducted our research seriously, there are still some limitations. (1) We retrieved documents from the WoSCC database and the Scopus database to enhance the persuasiveness of the conclusion. But there is an inevitable lack of information during data merging. In our merged data, all the information except the science categories has high integrity. Therefore, we refrain from analyzing the topic to avoid drawing misleading conclusions. (2) The study only includes English reviews and articles for analysis, which may affect the comprehensiveness of the analysis. (3) Bibliometrics is not absolute for the evaluation results of literature quality, such as citation analysis, which may be limited by time. It cannot wholly replace a systematic search. Nevertheless, our research remains highly valuable, providing guidance for future research on resistance to PD-1/PD-L1 inhibitors. However, the key breakthrough in drug resistance depends on further efforts by researchers.

## 5 Conclusion

Our study provides a comprehensive bibliometric analysis of research on PD-1/PD-L1 inhibitor resistance. It outlines the academic landscape, knowledge structure, current research hotspots, and classification of research models in this field. Key hotspots identified include the tumor microenvironment, biomarkers, and combination strategies. Bibliometric trends highlight growing interest in resistance mechanisms within the tumor microenvironment and biomarker-based patient stratification, both of which are increasingly investigated as potential approaches to inform personalized combination therapies for PD-1/PD-L1 inhibitor resistance. Several challenges remain. Combination therapies can increase toxicity, while the reliability of biomarkers is often limited. In addition, translating research findings into clinical practice has a low success rate. Overcoming these issues will require strengthened international collaboration, along with well-designed randomized controlled trials and organoid-based studies. Advanced approaches, including multi-omics analyses and artificial intelligence, may further help to address these obstacles.

## Data Availability

Publicly available datasets were analyzed in this study. This data can be found here: http://www.webofscience.com http://www.scopus.com/.
